# Is Staged Surgery Always Necessary for Schatzker Type IV–VI Tibial Plateau Fractures? A Comparison Study

**DOI:** 10.3390/life14060753

**Published:** 2024-06-13

**Authors:** Kai-Cheng Lin, Fu-Ting Huang, Chun-Yu Chen, Yih-Wen Tarng

**Affiliations:** Kaohsiung Veterans General Hospital, Kaohsiung 813, Taiwan; cychen@vghks.gov.tw (C.-Y.C.); ywtrang@vghks.gov.tw (Y.-W.T.)

**Keywords:** tibia plateau fracture, external fixation, open reduction and internal fixation, closed-incision negative-pressure therapy, negative-pressure wound therapy, outcome assessment

## Abstract

Aims: This study aims to compare the outcomes of immediate (followed by closed-incision negative-pressure therapy use) versus delayed ORIF in patients with Schatzker type IV–VI TPFs. Patients and Methods: A prospective study of patients undergoing ORIF between January 2018 and December 2019 was performed. The inclusion criteria were patients (>18 years) with a closed fracture sent to the emergency room (ER) within 24 h of injury. All the patients underwent preoperative image evaluation. Two senior orthopedic trauma surgeons evaluated the soft tissue condition in the ER by 5P’s of the compartment syndrome, judging the timing of the operation of definitive ORIF. Group 1 (*n* = 16) received delayed ORIF. Group 2 (*n* = 16) received immediate ORIF and ciNPT use. Patient follow-up occurred after 2 and 6 weeks and 3, 6, and 12 months after surgery. The assessments included the time to definitive fixation, the length of hospital stay, the time to bone union, surgical site complications, and reoperation within 12 months. A universal goniometer was used to measure the postoperative 3 m, 6 m, and 12 m ROM. Results: The patient demographics were similar between the groups (*p* > 0.05). Group 2 displayed significantly a shorter time to definitive fixation (5.94 ± 2.02 vs. 0.61 ± 0.28, *p* < 0.0001) and hospital stay (14.90 ± 8/78 vs. 10.30 ± 6.78, *p* = 0.0016). No significant difference was observed in the time to bone union, surgical site complication incidence, and reoperation rates (*p* > 0.05). Flexion and flexion–extension knee ROM were demonstrated to be significantly improved in Group 2, 3, 6, and 12 months postoperatively (*p* < 0.0001). Conclusions: In this study, early ORIF and ciNPT use resulted in a shorter hospital length of stay, a reduced time to early active motion of the knee, and improved knee ROM. These results suggest that early ORIF with ciNPT for Schatzker type IV–VI TPFs is safe and effective in some patients. However, further research to confirm these findings across larger and more diverse populations is needed.

## 1. Introduction

Tibial plateau fractures (TPFs) are fractures involving the articular surface of the proximal tibia, frequently associated with soft tissue injuries such as soft tissue crushing or contusion injuries [1]. These fractures are challenging, especially Schatzker type IV–VI fractures resulting from high-energy traumas [2,3]. A treatment dilemma exists for Schatzker type IV–VI TPFs because of high-energy traumas causing risky soft tissue conditions for the surgical fixation of these fractures. High-tension wound closure after fixation is a problem with which we are concerned. There are some reports showing that early definitive fixation can result in wound disruption, implant exposure, or skin necrosis, while delayed definitive fixation can result in joint stiffness of the knee [4,5,6]. Soft tissue swelling after acute fracture surgery can also pose a challenge, in that it may increase wound dehiscence, delay active motion of the joint, and increase the infection rate postoperatively.

Closed-incision negative-pressure therapy (ciNPT) has been previously reported to reduce the risk of wound dehiscence, surgical site complications, surgical site infection, seromas, and hematomas when applied over closed incisions compared to conventional post-surgical gauze dressings following high-risk lower-extremity fractures [7] and other orthopedic surgeries (i.e., hip and knee arthroplasty) [8,9,10,11]. Stannard et al. studied 249 patients with 263 high-risk fractures, including injuries to the tibial plateau, pilon, and calcaneus. They found that the relative risk of developing an infection was 1.9 times higher in the control patients than in the patients treated with NPWT [7].

Uncertainty exists between the clinical benefits of early or staged ORIF in Schatzker type IV–VI TPFs. The development and use of ciNPT in orthopedic procedures and high-energy fractures have suggested a potential role for the use of both early ORIF and ciNPT in the management of Schatzker type IV–VI TPFs. The aim of this study was to evaluate the safety and efficacy of treatment for early definitive open reduction and internal fixation (ORIF) in Schatzker type IV–VI TPFs with ciNPT use and compare the outcomes with staged treatment. We hypothesized that early definitive fixation of TPFs, performed by subspecialty-trained orthopedic trauma surgeons, and ciNPT use would result in satisfactory radiographic and functional outcomes, along with a reduced incidence of complications.

## 2. Patients and Methods

### 2.1. Patients

With the approval of the Institutional Review Board of the Kaohsiung Veterans General Hospital (IRB Number: VGHKS18-CT1-20), a prospective study of 32 patients with Schatzker type IV–VI TPFs undergoing ORIF in our level-one trauma center between February 2018 and December 2019 was performed. This study was performed in accordance with the 1964 Declaration of Helsinki and the relevant regulations of the US Health Insurance Portability and Accountability Act (HIPAA). For any adverse events, we had a team ready to receive emergency calls once patients had any kind of problems related to this study.

Thirty-two patients (eleven male patients and twenty-one female patients) ranging from 21 to 81 years of age were enrolled. The inclusion criteria were skeletally mature patients (over 18 years of age) with a closed fracture and patients sent to our emergency room within 24 h of injury. The exclusion criteria were polytraumas with multiple associated injuries, pathologic fractures, periprosthetic fractures, referred patients, and patients who could not complete follow-up for at least one year. Polytrauma patients, pathologic fractures, and periprosthetic fractures were excluded due to a possible bias when evaluating function and bone union, as they could have interfered with the data on the outcomes. Patients who had been referred or could not complete follow-up were excluded due to possible missing data. All the included patients provided informed written consent. The demographic information and fracture characteristics collected for this study included the following parameters: age, sex, smoking status, diabetes mellitus status, and fracture type (Table 1). 

### 2.2. Surgical Procedures

**Figure 1:**
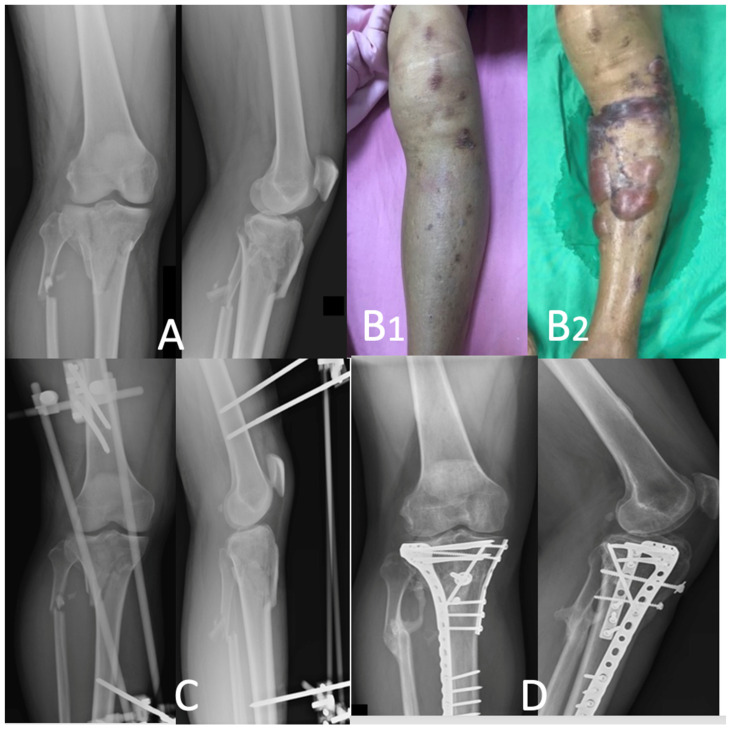
Typical case of bicondylar tibial plateau fracture with soft tissue swelling. A staged operation from external fixation to internal fixation is shown (**A**). A 44-year-old male sustained a Schatzker type VI tibial fracture. Plain films of comminuted proximal tibia fractures are demonstrated. Soft tissue swelling upon presentation is shown in (**B1**), and considerable bullous formation for suspicious compartment syndrome is shown in (**B2**). (**C**) Immediate stabilization with external fixation, waiting for the subsidence of soft tissue swelling, is performed (**D**). Definite fixation is completed with double plating 7 days after external fixation, with good healing of the fracture.

**Figure 2:**
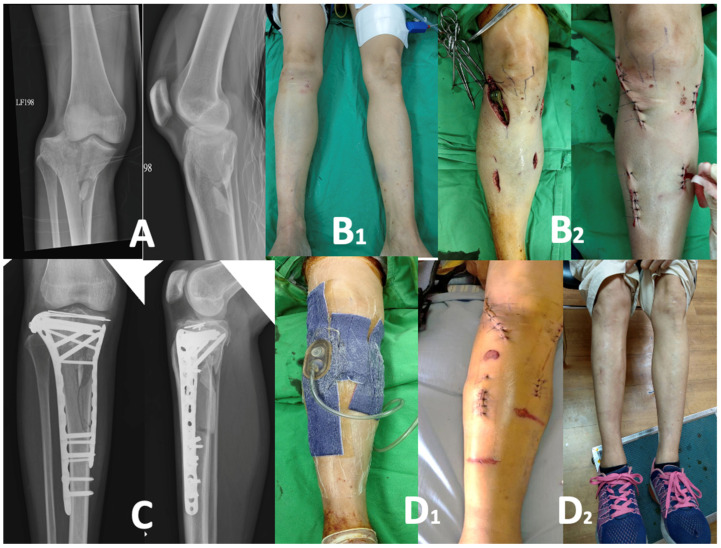
One-stage ORIF with plate tibia plateau fracture with dislocation. (**A**) A 38-year-old female sustained Schatzker type IV tibial plateau fracture with dislocation due to a scooter accident. Plain films of the injury are shown. (**B1**) At presentation, the soft tissue status is relative stable even though swelling has been found. (**B2**) ORIF is performed as a one-stage surgery. The wound used is shown above. (**C**) Plain films of ORIF are shown, with good reduction and fixation. (**D1**) Application of closed-incision negative-pressure therapy for the operative wound immediately postoperatively and the removal of the wound therapy device 5 days after its application. (**D2**) Right knee wound has healed nicely 6 months after ORIF.

All the patients were evaluated by one experienced team to plan for surgery. All the patients underwent X-ray and 3D computerized tomography (CT) for preoperative image evaluation for fracture classification and surgical planning. The patients were divided into two groups according to the availability dates of the orthopedic surgeons and the patients’ soft tissue conditions. The patients in Group 1 (*n* = 16) were treated with staged operations using a temporary bridging external skeletal fixator (ESF) with or without fasciotomy for 7~10 days. Definitive fixation was performed after soft tissue swelling subsidence or “wrinkle sign” presence as the current standard procedure (Figure 1A–D). The Group 2 patients (*n* = 16) were treated using a new protocol, with the early definitive fixation of the fracture as soon as possible. These patients underwent immediate ORIF to treat the fracture followed by the application of ciNPT over the closed incision (Figure 2A–D). 

All the fractures were fixed via anterolateral and posteromedial approaches, and dual-plate fixation (meaning lateral and medial buttress plating) was performed according to Luo’s three-column concept (meaning the lateral column, the medial column, and the posterior column, respectively). The bony defects of articular surface elevation were filled with a bone substitute [12]. A lateral-locking plate was applied to support the lateral split fragments or the depressed articular fragments. Fixed-angle multi-rafting screws were placed proximally in the subchondral bone to create a rafting effect. The medial anti-gliding buttress plate was applied over the medial site to provide medial support and avoid medial fragment subsidence. Finally, a posterior buttress plate was applied if the displaced posterior split fragment was found by shear force, to prevent loss reduction. Severe soft tissue swelling was anticipated after surgery due to extensive soft tissue dissection. 

For wound management, the Group 1 patients received ice packing and were prescribed appropriate deep-vein thrombosis prophylaxis. A dressing change was performed every day, and passive-motion therapy was arranged after swelling subsided. The patients in Group 2 received ciNPT (3M™ PREVENA^™^ Therapy, 3M Company, San Antonio, TX, USA) after surgery (Figure 2D), and continued negative pressure was applied at −125 mmHg. ciNPT was discontinued after 5 days, replaced by standard postoperative dressings. Active motion of the knee was encouraged to the greatest extent feasible, contingent upon patient symptoms. 

### 2.3. Postoperative Care and Follow-Up

After discharging the patients from the hospital, a routine follow-up was scheduled after 2 and 6 weeks, then 3, 6, and 12 months after the surgery, and annually thereafter. Fracture union was determined by radiographs taken immediately after the surgery and then at 6-week intervals until bone union was achieved. The post-surgical assessments included the time to definitive fixation (days), the length of hospital stay (days), the time to bone union, surgical site complications (SSCs), and secondary operation within 12 months. Bone union was defined by radiographic findings (anterior–posterior and lateral views), and complete bone union was confirmed when callus formation was observed in three or four cortices. 

### 2.4. Functional Evaluation

The knee injury and osteoarthritis outcome scores (KOOSs) [13] and the range of motion (ROM) of the knee (including extension and flexion) were evaluated and recorded during the postop 1-year follow-ups. The ROM was also assessed 3 and 6 months post surgery. 

### 2.5. Statistical Analysis

Data pertaining to the time to definitive fixation, the length of hospital stay, the time to bone union, the ROM, and the KOOS score (measured on a continuous scale) were collected. Statistical analyses were performed using the SAS Studio software release 3.8 (Cary, NC, USA). For the continuous variables, normality was assessed for each variable using a Shapiro–Wilk test. If the variable was not normally distributed, a two-sided Wilcoxon Rank-Sum test was performed, whereas a two-sided two-sample T-test was performed for normally distributed data. The categorical data were analyzed by Fisher’s exact test. For all the variables, statistical significance was determined at an alpha of 0.05.

## 3. Results

### 3.1. Patient Demographics

During this study, a total of 32 patients with an average age of 55.5 ± 13.2 years (range: 26–76 years) underwent ORIF surgery for TPFs. There were 16 patients in Group 1 (11 female and 5 male patients) and 16 patients in Group 2 (10 female and 6 male patients). Overall, the patient demographics, including age, sex, body mass index, smoking status, and percentage of patients with diabetes mellitus, showed no significant difference between the groups from the chi-square test. In addition, the percentage of fracture types (Schatzker type IV–VI) was similar between the two groups (as detailed in Table 1).

### 3.2. Surgical Outcomes

There was a significantly shorter time to definitive fixation for the patients in Group 2 (0.61 ± 0.28 days) compared to the Group 1 patients (5.94 ± 2.02 days, *p* < 0.0001, as detailed in Table 2). The Group 2 patients also had a significantly shorter hospital stay (10.3 ± 6.48 days) when compared with the Group 1 patients (14.9 ± 8.78 days, *p* = 0.0016, as detailed in Table 2). Although the time to fixation and the length of hospital stay were shorter for Group 2 (4.38 ± 1.02 days), the time to bone union was similar between the two groups (*p* = 0.56, as detailed in Table 2). 

The surgical site complications did not differ between the groups (*p* = 0.72, as detailed in Table 2), nor did the requirement for additional surgery within 12 months (*p* = 1.00, as detailed in Table 2). Four additional surgeries were required for the Group 1 patients, including three surgical debridement procedures due to poor wound healing and one implant removal due to implant failure. Three additional surgeries were required for the Group 2 patients, which included one related to the development of post-surgical vascular compromise, which required further vascular management, and two for local abscess removal around the incision sutures.

The flexion and flexion–extension knee ROMs were statistically significant between the two groups during the postoperative follow-ups, including those after 3 months, 6 months, and 12 months (*p* < 0.0001, as detailed in Table 3). The average KOOS score 12 months post surgery was higher for the Group 2 patients than for those in Group 1 (84.19 vs. 90.47, *p* < 0.0001). The KOOS scores, which measured the patient-reported outcomes related to knee pain, symptoms, and function, were notably higher for Group 2, suggesting better overall recovery and patient satisfaction post surgery. 

## 4. Discussion

The management of high-energy trauma with Schatzker type IV–VI tibial plateau fractures is challenging, as early definitive fixation can result in wound disruption, implant exposure, or skin necrosis, while delayed definitive fixation can result in joint stiffness of the knee [4,6]. Olszewski, Nathan MD, Manzano, and Givenchy MD et al. reported in their series that 101/1287 (7.8%) patients developed an infection. They found that temporary external fixation was a risk factor (OR 2.07; *p* = 0.013) associated with infection [14]. The current study evaluated the safety and efficacy of early definitive ORIF followed by ciNPT use in Schatzker type IV–VI TPFs compared to staged treatment outcomes. A significantly shorter time to definitive fixation and a shorter hospital length of stay were observed in the early-ORIF fixation group; however, the time to bone union was similar between the early-ORIF fixation and staged treatment groups. The surgical site complications did not differ between the two groups, nor did the requirement for additional surgery within 12 months. The knee ROM and the average KOOS score 12 months post surgery were significantly higher for the early-fixation group.

In the past, staged operations were the standard procedure in high-risk tibial plateau fracture surgeries [1,15]. Typically, a staged protocol featuring external fixation with or without fasciotomy would be performed initially for soft tissue injury concerns, but this increased the medical costs and the length of hospital stay. The time delay to definitive fixation was invariably dependent on the clinical signs for recovery of the soft tissue envelope and was ultimately a decision based on the experience of the orthopedic surgeons. Although the soft tissue was safe, the anatomic reduction technique became difficult in the articular surface, and knee joint stiffness was noted due to the extended immobilization time. Soft tissue swelling commonly occurred with the use of a spanning external fixator without fracture reduction. The capsule and ligament could remain swollen, and the immobilization could lead to a loss of elasticity and knee joint stiffness. Early ORIF avoids the use of an external fixator and may help in reducing the knee joint stiffness that is typically observed following delayed ORIF in TPFs [15]. The reduction in soft tissue swelling is easier in early ORIF due to the rigid fixation of the bony fragments [16].

The post-surgical management of the patients who underwent early ORIF in this study included the application of ciNPT, primarily to create an environment to hold the incision edges together and reduce postoperative wound edema. The previously published literature has reported significantly lower rates of wound dehiscence and total infections in patients who receive ciNPT over closed incisions compared to gauze dressings following high-risk lower-extremity fractures [7]. Although the effectiveness of ciNPT compared to other post-surgical dressings has not been assessed after surgical repair of Schatzker type IV–VI tibial plateau fractures, three randomized controlled trials and one prospective comparative study have reported reduced surgical site complications, including surgical site infections, seromas, and hematomas, when using ciNPT versus conventional post-surgical dressings for other types of orthopedic surgeries [8,9,10,11]. Similarly, Lin et al. recently reported good functional outcomes of 29 patients by applying ciNPT in acute-fracture surgery [17]. 

Positive clinical outcomes have been reported following the use of early ORIF after high-energy TPFs. Benirschke et al. reported excellent or satisfactory articular reduction (<2 mm) in 85.7% of their cases, along with no deep infection nor implant loosening, after immediate ORIF of 14 open Gustilo grade II and IIIA complex TPFs, although it should be noted that delayed primary closure after 5 days was used in these cases [18]. Similarly, Unno et al. reported good articular reduction (<2 mm) in 63.7% of their cases, with 15.7% of the TPFs needing additional surgery within 12 months, in a series of 102 patients with high-energy TPFs who underwent early definitive ORIF within 72 h of injury [19]. In our study, articular surface reduction in the early-ORIF group was easy without fragment adhesion; however, articular surface reduction in the delayed-ORIF group was difficult, requiring extensive soft tissue dissection to move the fragment adhesions. We summarize the studies regarding early versus delayed ORIF for tibial plateau fractures in Table 4. 

A previous study compared 25 patients with acute compartment syndrome related to type I–VI TPFs. Kim et al. reported no differences in the time to bone union, the percentage of complications, or the need for additional surgery in the patients managed with immediate internal fixation versus those treated with temporary external fixation [22]. In Kim et al.’s study on tibia fractures with compartment syndrome, they used NPWT for temporary soft tissue management after immediate ORIF, and there was no significant difference in bone union and complications. In our patients, early definitive ORIF for TPFs followed by ciNPT use significantly reduced the time to definitive fixation, the early active motion of the knee, and improved the ROM of the knee in flexion–extension motions in this complicated periarticular injury. The statistically significant higher flexion–extension ROM in Group 2 not only underscores the efficacy of early ORIF but also highlights its potential to enhance postoperative knee functionality, crucial for patients’ daily activities and overall recovery. Previous studies reported that the incidence of joint stiffness (ROM < 90°) was up to 7% after tibial plateau fractures, so the incidence of a decreased ROM (less than normal) of the knee joint was higher after surgery. Kim et al.’s study, there were no differences between the groups in the time to bone union, surgical site complications, or the need for additional surgery. The results support the idea that early ORIF followed by ciNPT use can be safe in some fractures [22]. In our study, there was a significant improvement in the ROM after early ORIF of TPFs and in people who did not receive external fixation [23,24]. The hospital length of stay and the knee ROM recovery time were shorter in the early-ORIF group than in the staged operation group. Li et al. [25] reviewed the studies on ciNPT application in traumas, including high-risk lower-extremity traumas (tibial plateau, pilon, and calcaneus), acetabular fractures, and hand traumas. All these studies showed significant improvements in surgical site infection and complications. They also mentioned, however, that only one prospective randomized study had been completed. Our study can demonstrate the good outcome of this new application in some cases. 

Although we observed excellent clinical outcomes with early definite ORIF and ciNPT use for Schatzker type IV–VI TPFs, we still hope for the time from injury to surgery to be as short as possible (i.e., less than 10 h) and for limited soft tissue dissection and gentle reduction to be required. The early surgical repair of TPFs is likely to be associated with an easier fracture reduction; however, the fragility of the soft tissue in these early stages might lead a surgeon to choose a more conservative approach to address the fracture. We still suggest staged operations in the presence of compartment syndrome, circumferential blisters, blood blisters, extensive soft tissue contusion or necrosis, or major trauma. 

As early ORIF may not be suitable for every patient, a thorough examination must be completed. In our study, one patient suffered from a delayed presentation of popliteal artery occlusion after immediate ORIF [26]. Fortunately, the leg was saved by emergent angioplasty with stents and a fasciotomy. The patient recovered with a fair outcome. Therefore, a thorough physical examination and a medical history assessment are important, especially for those patients who have suffered from a high-energy injury. 

The limitations of this study include the small sample size and the non-randomized nature of treatment group assignment. While the results are promising, the small sample size and the single-center design may limit the generalizability of these findings. Additionally, no definitive conclusions concerning the stability of fracture reduction can be drawn. While assessing the rate of reoperation after 12 months allowed for a comparison with other studies in the literature, it failed to allow for the assessment of the long-term consequences of early ORIF, such as the development of osteoarthritis. Moreover, the decision of whether to perform a staged operation or not is dependent on a surgeon’s experience, which could have been a bias in this study. Further studies utilizing a randomized controlled trial design or including a larger, more diverse patient population are expected and needed.

## 5. Conclusions

In this study, early ORIF followed by ciNPT use significantly shortened the hospital stays and improved the knee ROM in flexion–extension motions, demonstrating its efficacy compared to staged ORIF procedures. The results of this study suggest that the early surgical repair of TPFs generally facilitates an easier fracture reduction, particularly in patients without severe soft tissue injuries. These results support the adoption of early ORIF with ciNPT in routine clinical practice for Schatzker type IV–VI TPFs, potentially improving patient outcomes and reducing the healthcare costs associated with longer hospital stays and a delayed recovery. Future studies to define the criteria for early ORIF and the treatment protocols for Schatzker type IV–VI TPFs are warranted. 

## Figures and Tables

**Table 1:** Patient demographics and fracture characteristics.

Demographics	Group 1 (*n* = 16)	Group 2 (*n* = 16)	Overall (*n* = 32)
Age (years, mean ± SD)	56.8 ± 11.7	54.3 ± 14.8	55.5 ± 13.2
Sex (*n*, %)			
Male	5 (31.3)	6 (37.5)	11 (35.4)
Female	11 (68.7)	10 (62.5)	21 (64.6)
Body Mass Index (kg/m^2^, mean ± SD)	24.9 ± 3.91	25.6 ± 3.14	25.2 ± 3.50
Diabetes (*n*, %)	3(18.7)	2 (12.5)	7 (14.6)
Smoking Status (*n*, %)			
Never	12 (75.0)	14 (81.3)	26 (79.2)
Former	1 (6.3)	0 (0)	1 (2.1)
Current	3 (18.7)	3 (18.7)	6 (18.8)
Fracture Characteristics			
Cause of Injury (*n*, %)			
Traffic accident	15 (93.7)	15 (93.7)	30 (72.9)
Fall	1 (6.3)	1 (6.3)	2 (6.3)
Fracture Type			
IV	2 (12.5)	5 (31.3)	7 (25)
V	7 (43.7)	8 (50.0)	15 (50)
VI	7 (43.7)	3 (18.7)	10 (27.1)
Articular Depression >10 mm (*n*, %)	11 (68.7)	11 (68.7)	22 (89.8)
Displacement > 3 cm (*n*, %)	7 (43.7)	8 (50.0)	15 (46)
Dislocation (*n*, %)	5 (31.3)	8 (50.0)	13 (41.2)
Implant Removal (*n*, %)	4 (25.0)	0 (0)	4(7.7)

SD = standard deviation.

**Table 2:** Surgical and post-surgical outcomes.

Outcomes	Group 1 (*n* = 16)	Group 2 (*n* = 16)	*p*-Value
Time to Definitive Fixation (days, mean ± SD)	5.94 ± 2.02	0.61 ± 0.28	<0.0001 ^A^
Length of Stay (days, mean ± SD)	14.9 ± 8.78	10.3 ± 6.48	0.0016 ^A^
Time to Union (months, mean ± SD)	4.56 ± 2.28	4.38 ± 1.02	0.56 ^A^
Surgical Site Complications (*n*, %)			
Yes	4 (25.0)	3 (19.8)	0.72 ^B^
No	12 (75.0)	13 (80.2)	
Additional Surgery (*n*, %)			
Yes	4 (25.0)	2 (12.6)	1.00 ^B^
No	12 (75.0)	14 (77.4)	

^A^ Wilcoxon Rank-Sum test; ^B^ Fisher’s exact test; and SD = standard deviation.

**Table 3:** Range of motion and patient-reported outcome scores.

Outcome	Group 1 (*n* = 16)	Group 2 * (*n* = 16)	*p*-Value
Extension (mean ± SD)			
3 month	3.13° ± 4.03°	4.69° ± 4.91°	0.326 ^A^
6 month	3.13° ± 4.03°	5.31° ± 4.91°	0.145 ^A^
12 month	3.13° ± 4.03°	5.00° ± 4.91°	0.227 ^A^
Flexion (mean ± SD)			
3 month	96.56° ± 8.51°	115.31° ± 6.21°	<0.0001 ^A^
6 month	110.31° ± 11.61°	128.75° ± 6.60°	<0.0001 ^A^
12 month	110.31° ± 11.61°	128.67° ± 6.81°	<0.0001 ^A^
Flexion–Extension (mean ± SD)			
3 month	93.44° ± 9.44°	110.63° ± 8.30°	<0.0001 ^A^
6 month	107.19° ± 11.10°	123.44° ± 8.18°	<0.0001 ^A^
12 month	107.19° ± 11.10°	123.67° ± 8.40°	<0.0001 ^A^
KOOS (mean ± SD)			
12 month	84.19 ± 3.87	90.47 ± 3.55	<0.0001 ^B^

^A^ Wilcoxon Rank-Sum test; ^B^ two-sample *t*-test; SD = standard deviation; KOOS = knee injury and osteoarthritis outcome score; and * only 30 patients completed the 12 month follow-up.

**Table 4:** Summary of publications of early vs. delayed ORIF for tibial plateau fractures.

Authors. (Year)	Study Design and Group	Outcomes	Limitations
Benirschke (1992) [18]	Prospective, 14 open type V, VI TPF, immediate ORIF after deb.	HSS: 81.5 Knee Score: 84.6. 10: excellent radiographic grade.	Only included open fractures.
Xu (2013) [20]	Retrospective, 5 surgical groups. 125 patients.	Optimal surgical timing is 4 hours. Overall wound complication incidences were 20.0%, 41.6%, 33.3%, 2.5%, and 16.7% within 4 hours, 4 hours to 3 days, 3-5 days, 5-8 days, and more than 8 days after injury. Failed fixation was clearly observed in Group 1 (23.1%, 6/26) and Group 5.	10.4% loss of follow up. small number in specific group
Unno (2017) [19]	Retrospective, 102 TPFs, fixation <72 hrs.	Nonstaged: 91.3% Sixteen (15.7%) required additional surgeries. SF-36 (12 months): 42.6	Retrospective review by prospective data collection. No strictly defined criteria were used to direct the choice between primary ORIF and the staged protocol, which was made by the treating surgeon.
Mesa (2024) [21]	Retrospective, 186 TPFs, aORIF (acute) vs. sORIF (staged).	aORIF was associated with a significantly lower rate of superficial infection (*p* = 0.01), arthroplasty (*p* = 0.003), and unplanned reoperation (*p* = 0.005). No increased risk of complications with aORIF.	aORIF or sORIF was selected by the surgeons.

## Data Availability

The original contributions presented in the study are included in the article, further inquiries can be directed to the corresponding authors.
